# Impact of Musculoskeletal Limitations on Cardiac Rehabilitation Participation

**DOI:** 10.3389/fcvm.2021.688483

**Published:** 2021-06-28

**Authors:** Marta Supervia, Jose R. Medina-Inojosa, Carmen M. Pérez-Terzic, Saurabh Sharma, Kashish Goel, Kristin Vickers Douglas, Karen Salz, Randal J. Thomas

**Affiliations:** ^1^Gregorio Marañón General University Hospital, Gregorio Marañón Health Research Institute, Madrid, Spain; ^2^Cardiovascular Rehabilitation Program, Department of Cardiovascular Diseases, Mayo Clinic, Rochester, MN, United States; ^3^Department of Physical Medicine and Rehabilitation, Mayo Clinic, Rochester, MN, United States; ^4^Guthrie Robert Packer Hospital, Sayre, PA, United States; ^5^Department of Cardiovascular Diseases, Vanderbilt University Medical Center, Nashville, TN, United States; ^6^Department of Psychiatry and Psychology, Mayo Clinic, Rochester, MN, United States

**Keywords:** cardiovascular rehabilitation, patient participation, musculoskeletal diseases, musculoskeletal pain, cardiac rehabilitation, physiatrist

## Abstract

**Background:** To help clarify a potential barrier to cardiac rehabilitation (CR) participation we sought to examine the association between musculoskeletal limitations (MSLs) and CR enrollment and participation.

**Methods:** Consecutive CR eligible individuals hospitalized for a cardiac event (myocardial infarction, percutaneous coronary intervention, and/or coronary artery bypass graft) between the months of November 2007 and May 2008, were asked to complete a mailed survey within 2 weeks after hospital discharge, assessing demographic factors, Patient Health Questionnaire (PHQ-9), participation in CR and MSLs through a validated MSLs screening tool. CR enrollment rates were compared between patients with and without MSLs.

**Results:** Three hundred and twenty-one (37%) of patients contacted responded to our survey, including 228 males (71%), with a mean age 68 ± 10.8 years, of whom 98% were Caucasian. Eighty-two percent of responders reported a musculoskeletal disorder at the time of hospital discharge. Arthritis was the most frequent diagnosis (45%). Muscle or joint pain sufficient to limit the ability to do moderate exercise was reported in 52% of the respondents. Problems with balance affected 37%, of whom 45% reported a fall within the previous year. No significant difference in CR enrollment was observed in respondents with and without MSLs [OR = 0.98, 95% CI (0.88–1.09), *p* = 0.750]. Similar results were found when severity and number of MSLs were taken into account. However, we found that when compared to those without MSLs, the presence of MSLs was associated with lower CR participation (OR = 0.80, 95%, CI: 0.65–0.97, *p* = 0.0252).

**Conclusion:** Despite a high prevalence of MSLs among CR-eligible patients, we found no association between MSLs and CR enrollment. However, patients with MSLs attended significantly fewer CR sessions as compared to patients without them. CR programs should consider providing additional support and interventions to patients with MSLs in order to optimize their adherence to prescribed CR sessions.

## Introduction

Cardiac rehabilitation (CR) is a cost-effective and integral component of the continuum of care for cardiac patients (1, 2) that improves functional status and psychological health (1), and reduces morbidity, re-hospitalization and mortality rates (3). CR that was later established in clinical practice guidelines as a Class I recommendation (4, 5) for patients with a wide range of cardiac conditions. However, despite these facts, CR is underutilized 2, particularly in patients with significant comorbidities (6, 7). A number of potential factors have been identified as barriers to CR participation, including patient-, medical-, and healthcare system-level factors.

Musculoskeletal limitations (MSLs) are common in the general population and particularly prevalent in patients with coronary artery disease (8, 9). They are a major source of disability, immobility and dependence (10–13), yet little is known about their potential impact on CR participation.

Since it is unknown if MSLs prevent patients from enrolling in a CR program, we aim to examine (1) the prevalence of MSLs in patients referred to CR and (2) explore its possible roles as a barrier to patient enrollment and completion in phase II CR.

We sought to help clarify whether or not MSLs serve as a barrier to CR participation by assessing, in a consecutive sample of hospitalized patients, the association between MSLs and CR enrollment, as well as between MSLs and CR participation.

## Materials and Methods

Consecutive individuals hospitalized for a CR-eligible cardiac event [myocardial infarction (MI), percutaneous coronary intervention (PCI), and/or coronary artery bypass graft (CABG) surgery] between November 2007 and May 2008 were mailed a study packet containing a consent form and survey within 2 weeks of hospital discharge.

Individuals were identified through daily census records of the Mayo Clinic hospitals in Rochester, Minnesota, by the inpatient Cardiovascular Health Clinic team. Patients were included in the study if they were >18 years of age and had one or more of the qualifying diagnoses (MI, PCI, and/or CABG). Patients who were unable to read and write in English were excluded. Individuals who met the study's entry criteria were sent a study packet containing a written consent form, as well as a detailed description of the study, and the study survey tool. Individuals who agreed to enter the study were instructed to fill out the consent form and the study survey. Persons who did not agree to enter the study were instructed to fill out the consent form indicating that they did not wish to participate in the study.

Self-report items were used to assess participants' demographic information (age, gender, education level, marital status, ethnicity, employment status), self-reported CR recommendation by health care provider, depressive symptoms, presence of comorbidities (Charlson index), perceived health (5-point scale ranging from “poor” to “excellent”), and presence of MSLs as documented by a validated Musculoskeletal Limitations Screening questionnaire. Enrollment and number of sessions attended in CR were obtained from the medical records.

Depressive symptoms were assessed with the Patient Health Questionnaire (PHQ-9), a self-report measure that captures both cognitive and physical symptoms of clinical depression with a higher score indicating more depressive symptoms. Scores range from 0 to 27, with higher scores indicating greater depressive symptom severity. Scores of 10 and higher indicate moderate to severe depressive symptom severity (14).

### Identification of Musculoskeletal Limitation

We used a 7 item screening tool that was developed in the Cardiovascular Health Clinic to identify MSLs that may impact a person's quality of life and their ability to exercise or participate in other related cardiovascular disease prevention activities. Seven questions in the screening tool asked about the (1) intensity of the muscle and joint pain on a scale of (1–10); (2) duration of pain; (3) effect of existing pain on the ability to perform moderate exercises; (4) pain relief achieved by medication usage; (5) effect of balance problems on the ability to perform moderate exercises; (6) presence of specific musculoskeletal diagnoses (arthritis, spinal stenosis, herniated vertebral disc, etc.); and (7) history of any of the following: a fall in the previous year, an amputation of a limb/finger/toe, joint replacement surgery, any joint surgery (including back) and stroke (Supplementary Table 1). Test-retest validity of the musculoskeletal screening tool was assessed and showed high correlation (see Supplementary Table 1 and Table 1). This study was approved by Mayo Clinic Institutional Review Board.

**Table 1:** Demographics.

**Variable**	**Total cohort 321**	**Men 228 (71%)**	**Women 93 (29%)**	***P-*value**
Age—(years)	68 ± 10.8	67.8 ± 10.5	69.7 ± 11.6	0.6
Body mass index	29.1 ± 5.2	29.5 ± 6.6	28.9 ± 4.6	0.4
**Smoking status**				
Yes	17 (5.4%)	13 (6%)	4 (5.0%)	0.6
No	300 (94.6%)	212 (94%)	88 (95%)	
Depression symptoms (PHQ-9 score)	5.17 ± 4.5	4.7 ± 4.6	6.3 ± 4.3	0.008
Education levels (years)	13.9 ± 3.5	14.0 ± 3.6	13.9 ± 3.1	0.6
**Marital status**				
Married	242 (75.4%)	193 (84.6%)	49 (52.7%)	<0.001
Widowed	31 (9.7%)	10 (4.4%)	21 (22.6%)	
Separated	2 (0.65%)	1 (0.4%)	1 (1.1%)	
Divorced	32 (9.9%)	20 (8.8%)	12 (12.9%)	
Not married	12 (3.7%)	4 (1.7%)	8 (8.6%)	
Not married but living with a partner	2(0.6%)	0	2 (2.1%)	
**Ethnicity**				
White, non-Hispanic	314 (97.8%)	222 (97.4%)	92 (98.9%)	0.15
Black, non-Hispanic	1 (0.3%)	0	1 (1.1%)	
Asian or Pacific Islander	0	0	0	
American Indian/Alaskan Native	2 (0.6%)	2 (0.9%)	0	
Hispanic	1 (0.3%)	1 (0.4%)	0	
Other	3 (0.9%)	3 (1.3%)	0	
Perceived health^*^				
Excellent	10 (3.1%)	5 (2.2%)	5 (5.3%)	0.48
Very good	66 (20.7%)	50 (22.2%)	16 (17.2%)	
Good	174 (54.7%)	122 (54.2%)	52 (55.9%)	
Fair	57 (17.9%)	41 (18.2%)	16 (17.2%)	
Poor	11 (3.5%)	7 (3.1%)	4 (4.3%)	
Recent recommendation by a health care provider for CR participation^*^				
Yes	264 (86.0%)	188 (84.7%)	76 (89.4%)	0.69
No	43 (14%)	34 (15.3%)	9 (10.6%)	
Charlson index category^*^				
0	114 (35.5%)	82 (35.9%)	32 (34.4%)	0.003
1–2	110 (34.3%)	73 (32%)	37 (39.8%)	
3–4	48 (14.9%)	34 (14.9%)	14 (15.1%)	
≥5	49 (15.3%)	39 (17.1%)	10 (10.7%)	

*SD, standard deviation*.

* *Based on data available*.

### Statistical Analysis

We assessed study variables as frequencies and percentages, mean values and standard deviations (SD) or median and interquartile ranges, depending on the distribution of the variables. We calculated intra-class correlation indices and Kappa statistic to test-retest validity of the musculoskeletal screening tool.

Multivariate logistic regression analysis was used to assess independent predictors associated with CR enrollment adjusting for age, gender, smoking status, PHQ-9 score, level of education, musculoskeletal comorbidities, and the Charlson comorbidity index (**Table 4**). Findings were summarized using odds ratios (OR) and 95% confidence intervals. In all cases, two-tailed *p*-values ≤ 0.05 were used to denote statistical significance α level of 0.05. All analyses were completed using SAS® 9.3 (SAS Institute Inc., Cary, NC).

## Results

### Response Rate and Participants Characteristics

Out of 862 patients who were sent study packets, 321 (37%) consented to participate and returned the study survey.

The sample composition was predominately male (71%), white (98%), married (75.4%), with a relatively high level of education (13.9 years ± 3.5), and with a mean age of 68 ± 10.8 years. The mean PHQ9 score was 5.2 ± 4.5, with women having higher scores than men (6.3 ± 4.3 vs. 4.7 ± 4.6, *p* = 0.008). Eighty-six percent of the patients reported a recent recommendation by a health care provider to participate in CR. Almost 80% of patients perceived their health status to be “good” or better, and over 70% were classified in the Charlson Index categories 0–2 (Table 1). Enrollment in CR was reported in 54% (56% of men and 53% of women).

### Prevalence of Musculoskeletal Limitations

Musculoskeletal limitations including any type of chronic muscular or joint pain of varying intensity and duration, were present in 82% of respondents (83.3% male, 85.2% female), with over 54.2% noting that the MSL had been present for at least 1 year. Sixty percent of patients reported that MSL-related pain limited their ability to perform moderate exercises such as walking, biking or swimming. Balance problems were reported by 37% of respondents (32.9% in men and 47.1% in women, *p* = 0.01), while 53% reported a fall in the previous year. Gender differences were found in joint swelling (8.3% in men vs. 19.3% in women, *p* = 0.007) and spinal stenosis (3.1% in men and 14% in women, *p* = 0.0005) too (Table 2). Fifty-five percent of respondents reported having at least one MSL-related diagnosis including: arthritis in the joints (45.2%), joint swelling (17%), inflammatory arthritis (13%), and previous joint surgery (20%) (see Table 2). Women were more frequently diagnosed with any or all of the possible MSL-related diagnoses (65.59% women vs. 50.88 men, *p* = 0.01).

**Table 2:** Patient distribution according to musculoskeletal limitations screening questionnaire.

	***N* (321)**	**Men (228)**	**Women (93)**	***P-*value**
**Intensity of muscle/joint pain**	302	214	88	
No pain	51 (16.9%)	38 (17.7%)	13 (14.8%)	0.14
Pain rated 1–5	175 (58.3%)	129 (60.3%)	46 (52.3%)	
Pain rated 6–10	76 (23.7%)	47 (22%)	29 (32.9%)	
**Duration of joint pain**	310	221	89	
No pain	83 (26.7%)	63 (28.5%)	20 (22.5%)	0.16
<1 month	21 (6.8%)	17 (7.7%)	4 (4.5%)	
1 month−1 year	38 (12.2%)	29 (13.1%)	9 (10.1%)	
>1 year	168 (54.2%)	112 (50.7%)	56 (62.9%)	
**Effect of pain on ability to perform moderate exercises (walking, biking, or swimming)**	310	220	90	
No limitation	125 (40.3%)	100 (45.4%)	25 (27.8%)	0.01
Slight to moderate limitation	161 (52%)	105 (47.7%)	56 (62.2%)	
Severe limitation	24 (7.7%)	15 (6.8%)	9 (10%)	
**Pain relief with medications**	278	198	80	
No pain medication	138 (49.2%)	106 (53.5%)	32 (40%)	0.07
Pain medications present	140 (50.4%)	92 (46.5%)	48 (60%)	
No pain relief	10 (3.6%)	8 (4%)	2 (2%)	
Slight to near total pain relief	130 (46.8%)	84 (42.4%)	46 (57.5%)	
**Balance problems affecting ability to perform moderate exercise**	309	222	87	
No balance problems	195 (63.1%)	149 (67.1%)	46 (52.9%)	0.01
Balance problems present	114 (36.9%)	73 (32.9%)	41(47.1%)	
No limitation	41 (13.3%)	27 (12.2%)	14 (16.1%)	
Slight to moderate limitation	65 (21%)	43 (19.4%)	22 (25.3)	
Severe limitation	8 (2.6%)	3 (1.3%)	5 (5.7%)	
**Patients who were told by a healthcare professional that they have the following**	177	116	61	
Arthritis in the joints	145 (45.2%)	92 (40.3%)	53 (57%)	0.006
Herniated disk in the back/neck	45 (14%)	29 (12.7%)	16 (17.2%)	0.3
Inflammatory arthritis	24 (7.5%)	14 (6.1%)	10(10.7%)	0.1
Osteoporosis	28 (8.7%)	5 (2.2%)	23 (24.7%)	<0.001
Spinal stenosis	20 (6.2%)	7 (3.1%)	13 (14%)	0.0005
Vertebral or spinal fracture	11 (3.4%)	7 (3.1%)	4 (4.3%)	0.5
Joint swelling	37 (11.5%)	19 (8.3%)	18 (19.3%)	0.007
Neurological problem other than stroke	6 (1.9%)	4 (1.7%)	2 (2.1%)	0.8
**Patients with following history**	121 (37.7%)	87 (38.2%)	34 (36.6%)	0.7
Falls in past year	69 (21.5%)	52 (22.8%)	17 (18.3%)	0.3
Amputation of a limb/finger/toe	6 (1.8%)	5 (2.2%)	1 (1.1%)	0.4
Joint replacement surgery	29 (9%)	19 (8.3%)	10 (10.7%)	0.4
Joint surgery (including back)	32 (10%)	22 (9.6%)	10 (10.7%)	0.7
Stroke	21 (6.5%)	13 (5.7%)	8 (8.6%)	0.3

### Predictors of CR Enrollment

Univariate predictors of CR enrollment are shown in Table 3 and include: age, gender, smoking current status, PHQ-9, education level, Charlson index and MSLs screen. Respondents with MSLs were no more likely to enroll in CR than those without MSLs (77.38 vs. 77.78%, *p* = 0.9). This was not different when stratifying the analysis by gender, for males *p* = 0.5, for females *p* = 0.6.

**Table 3:** Predictors of CR participation.

**Parameter**	**Odds ratio**	**95% CI lower-upper**	***P-*value**
Age	0.97	0.95–0.99	0.04
Male	0.9	0.5–1.4	0.6
Smoking	0.6	0.21–1.7	0.3
PHQ-9	0.96	1.01–1.03	0.02
Health care provider recommendation	2.67	1.37–5.44	0.0036
Education level	1.2	1.05–2.62	0.04
Charlson category 2	0.80	0.42–1.54	0.5
Charlson category 3	0.47	0.25–0.85	0.01
Charlson category 4	0.84	0.46–1.57	0.59
MSLs screen	0.97	0.57–1.66	0.9

*Univariate analysis*.

Multivariate analysis revealed that a recommendation by a health care provider for the patient to participate in CR was the strongest independent predictor of CR enrollment (HR 3.62, 95% CI: 1.55–8.47, *p* = 0.002). In addition, a lower PHQ9 score was independently predictive of CR enrollment compared to those with a higher score (HR 0.93, 95% CI: 0.87–0.99, *p* = 0.016). Younger age also predicted CR enrollment (HR 0.96, 95% CI: 0.94–0.99, *p* = 0.005) (see Table 4). Presence of at least one MSL was not predictive of CR enrollment, and this finding did not change even after consideration of the number and severity of MSLs [OR = 0.97, 95% CI (0.57–1.56), *P* = 0.9—any MSL vs. no MSL]. Respondents with severe MSLs were no more likely to enroll in CR than were respondents with mild and no MSLs (HR 0.98, 95% CI: 0.88–1.09 *p* = 0.75).

**Table 4:** Predictors of CR participation.

**Parameter**	**Estimate**	**Standard error**	**Odds ratio**	**95% CI lower-upper**	***P-*value**
Age	−0.03	0.014	0.96	0.94–0.99	0.005
Male	−0.14	0.31	0.87	0.47–1.60	0.651
Smoking	0.18	0.62	1.20	0.35–4.06	0.771
PHQ-9	−0.07	0.031	0.93	0.87–0.99	0.016
Health care provider recommendation	1.28	0.43	3.62	1.55–8.47	0.002
Education level	0.008	0.04	1.01	0.93–1.10	0.842
Charlson category 2	−0.51	0.33	0.60	0.32–1.13	0.116
Charlson category 3	−0.50	0.41	0.61	0.27–1.35	0.221
Charlson category 4	0.70	0.47	2.01	0.81–5.02	0.133
MSLs screen	−0.02	0.0536	0.983	0.885–1.092	0.750

*Multivariate analysis*.

### Predictors of CR Attendance

Data on the number of CR sessions attended per patient were available in the 79 patients who lived in the Rochester, Minnesota area and who attended the Mayo Clinic CR Program, while session attendance was not available on 111 patients who were referred to other CR centers outside of the Rochester area. Among the patients who enrolled in the Mayo Clinic CR program, a lower pain score with activity was associated with a higher number of sessions patients attended (OR = 1.20, 95%, CI: 1.05–1.50, *p* = 0.02026). Presence of pain over 1 month or the inability to do exercise because of pain was associated with a lower CR attendance (OR = 0.18, 95% CI: 0.04–0.64; OR = 0.25, 95% CI: 0.09–0.63). Of interest, balance problems were more frequently reported among participants who attended more CR sessions (OR = 3.02, 95% CI: 0.12–0.87). However, we found that when compared to those without MSLs, the presence of MSLs was associated with lower CR participation (OR = 0.80, 95%, CI: 0.65–0.97, *p* = 0.0252).

## Discussion

While MSLs were commonly present in our study cohort (82%), they were not associated with lower CR enrollment, even after taking into consideration the number and severity of MSLs that were reportedly present. Factors that were predictive of CR enrollment included a recommendation by a health care provider for the patient to participate in CR, younger age, and lower depressive symptoms (i.e., lower PHQ-9 score). While CR enrollment rate was 54%, the likelihood of CR enrollment decreased in stepwise fashion with older age (*p* = 0.005) and higher PHQ 9 score, similarly in men and women (*p* = 0.69). An individual receiving a strong health care provider's recommendation to attend CR was 4 times as likely to enroll in CR compared to individuals who did not receive such a recommendation. Overall, we found that MSLs were not associated with lower enrollment in a CR program (Table 4), but they were associated with lower CR session attendance.

These findings are important for several reasons. First, MSLs are common in individuals with cardiovascular disease, suggesting the need for CR programs and other health care providers to screen for and appropriately address the MSLs that are present in their patients. While we did not find MSLs to be a barrier to CR enrollment, we did find that MSLs are associated with lower attendance at CR sessions. Furthermore, MSLs have been previously reported to occur during CR or may be exacerbated during CR (9, 15), which might help explain why MSLs are associated with lower attendance at CR sessions. Close collaboration between CR and physical medicine and rehabilitation professionals appears to be warranted for patients with MSLs who participate in CR. The use of personalized exercise prescriptions in such patients could potentially help them improve functional capacity during CR9 (16–21), as well as CR attendance.

Another reason our findings are important is because they reinforce previous reports that have found a high prevalence and accelerated increase of incidence (22) of comorbidities (two or more chronic health conditions) in individuals with cardiovascular disease (CVD), particularly with regards to the co-existence of musculoskeletal conditions (8, 9). This may be secondary to shared risk factors for both MSLs and CVD, such as obesity and aging of the population (23–25). Our study is similar to previous studies that have also shown a high prevalence of MSLs among CR-eligible patients (8, 9). Other studies have also found that comorbidities increase the complexity of care required for patients with CVD, can be an important impediment to patient adherence, functional status, and quality of life (23, 26–28), and are associated with higher healthcare resource utilization (29)^1^. Care coordination between CR professionals and others involved in the care of CR patients is crucial in patients with comorbid conditions (26, 28, 30), so that those conditions can be identified and managed effectively, reducing the potential for fragmentation of care which can result in the care of patients with multiple, complex comorbidities.

Finally, our findings are important because they help clarify whether or not MSLs serve as a barrier to CR enrollment, an issue that is both misunderstood and understudied (4, 7, 31–35). We found that while MSLs are common and symptomatic in CR patients, they are not associated with lower CR enrollment, even when the number and severity of MSLs are considered. However, we did find that MSLs are associated with lower CR adherence, suggesting that the careful assessment and management of MSLs are important in optimizing the potential cardiovascular benefits of CR in participating patients with MSLs.

### Study Limitations

Our study is limited by the fact that our data are from one center in one geographic area of the mid-western United States with a largely white population. Thus, our findings may not be generalizable to other centers and other populations. In addition, the high prevalence of MSLs may limit our ability to assess the true impact of MSLs on CR enrollment. However, we found no association between MSLs and CR enrollment even when restricting our definition of MSLs to a smaller group of CR patients with the highest number and severity of MSLs. Our data are based on self-reported surveys, which is a potential limitation to our study. The MSL screening tool used, however, was found to have high test-retest validity, which adds support for the use of the self-reported tool in identifying CR-eligible patients with MSLs. The response rate to our survey was moderate (37% of invited participants), which may have resulted in a sampling bias that could have led to patients with MSLs being more likely to complete and return the survey. However, we sought to overcome this possible bias by considering in our analyses the number and severity of reported MSLs. We found that in all analyses, MSLs were not associated with CR enrollment. Finally, our analyses regarding attendance at CR sessions were limited data from patients in our cohort who attended CR at the Mayo Clinic and did not include data from patients from other regions of the country who received their initial cardiovascular care at the Mayo Clinic but were subsequently referred to a CR program in other regions of the country, near where they lived.

## Conclusion

We found that MSLs, while common and significantly symptomatic in our cohort of CR-eligible patients, were not associated with lower CR enrollment, but were associated with lower CR attendance. MSLs are an important and common comorbidity in CR patients, requiring close collaboration and care coordination between CR staff and physical medicine and rehabilitation professionals to optimize CR care and outcomes in patients with MSLs (36). Additional research is warranted to identify the most effective management strategies for this common and important group of CR patients.

## Data Availability Statement

The original contributions presented in the study are included in the article/Supplementary Materials, further inquiries can be directed to the corresponding author/s.

## Ethics Statement

The studies involving human participants were reviewed and approved by Mayo Clinic Institutional Review Board. The patients/participants provided their written informed consent to participate in this study.

## Author Contributions

MS, JM-I, CP-T, SS, KG, KV, KS, and RT: conceptualization, writing—review, and editing. JM-I, SS, and KG: formal analysis. CP-T, SS KG, KV, KS, and RT: data curation. MS: writing—original draft preparation. RT: supervision. All authors have read and agreed to the published version of the manuscript, gave final approval, and agree to be accountable for all aspects of work ensuring integrity and accuracy.

## Conflict of Interest

The authors declare that the research was conducted in the absence of any commercial or financial relationships that could be construed as a potential conflict of interest.

## Footnotes

^1^http://www.cdc.gov/chronicdisease/resources/publications/aag/arthritis.htm
