# Supplementary material for: Impact of Musculoskeletal Limitations on Cardiac Rehabilitation Participation
Source: Front Cardiovasc Med. 2021 Jun 28;8:688483. doi: 10.3389/fcvm.2021.688483 (PMC8273239; doi:10.3389/fcvm.2021.688483)
Supplement: Supplementary file 1 [file Table_1.docx]

Supplementary Material

# Supplementary Tables

**Supplementary Table 1**. Test-retest validity of the musculoskeletal screening

| **Question** | **OR** | **CI** | **P value** |
| --- | --- | --- | --- |
| 1. How would you rate the amount of muscle and/or joint pain you have with activity?  \| 0 \| 1 \| 2 \| 3 \| 4 \| 5 \| 6 \| 7 \| 8 \| 9 \| 10 \| \| --- \| --- \| --- \| --- \| --- \| --- \| --- \| --- \| --- \| --- \| --- \| \| No pain \| \| \|  \| Moderate pain \| \| \|  \|  \| Extreme pain \| \| | 0.68478* | 0.44-0.93 | p<0.05 |
| 2. How long have you had muscle and/or joint pain? (Check the **one** answer that applies best to you)  I do not have any muscle and/or joint pain  Less than 1 month  Between 1 month and 3 months  Between 3 months and 6 months  Between 6 months and 1 year  Greater than 1 year | 0.77542* | 0.60-0.95 | p<0.05 |
| 1. How much does muscle or joint pain affect your ability to do moderate exercise, such as walking, biking, or swimming?   No limitation (can do whatever amount of moderate exercise I desire)  Slight limitation  Mild limitation  Moderate limitation  Severe limitation (can do little or no moderate exercise) | 0.85246* | 0.73 -0.98 | p<0.05 |
| 1. If you are taking medication to relieve muscle and/or joint pain, how would you describe the amount of pain relief the medication gives you?   Almost no pain relief  Slight pain relief  Moderate pain relief  Total or near-total relief  I don’t take pain medication | 0.38509* | 0.016-0.75 | p<0.05 |
| 1. How much do balance problems affect your ability to do moderate exercise, such as walking, biking, or swimming:   (Check the **one** answer that applies best to you)  I don’t have any balance problems  No limitation (I have balance problems but can do exercise without limitations)  Slight limitation  Mild limitation  Moderate limitation  Severe limitation (can do little or no exercise due to balance problems) | 0.88776* | 0.80-0.98 | p<0.05 |
| 1. Have you ever been told by a healthcare professional that you have any of the following conditions? (Check **all** answers that apply to you)   Arthritis in the joints  Herniated disk in the back or neck  Inflammatory arthritis (rheumatoid arthritis, lupus, etc.)  Osteoporosis  Spinal stenosis  Vertebral or spinal fracture  Joint swelling  Neurological problem other than stroke (like Parkinson’s Disease, for example) | 0.8991** | 0.01- 1.28 | P=0.3 |
| 1. Have you ever had any of the following?   A fall sometime during the past year  Amputation of a limb, finger, or toe  Joint replacement surgery  Joint surgery (including back)  Stroke | 0.6452** | -0.11 -0.02 | P=0.3 |

*CCC represents interclass correlation **Kappa
